# Anticancer Effects of *Ziziphus jujuba* in Human Breast Carcinoma: An Integrated Study of Network Pharmacology, Molecular Docking and Dynamics Simulations and in-vitro Experimental Validation

**DOI:** 10.5812/ijpr-169917

**Published:** 2026-04-20

**Authors:** Jinxin Guo, Jing Shen

**Affiliations:** 1 Department of Pharmacy, Sijing Hospital, Songjiang District, Shanghai, China

**Keywords:** *Ziziphus jujuba*, Network Pharmacology, Molecular Docking, Molecular Dynamics, Bioassays, Phytochemicals

## Abstract

**Background:**

*Ziziphus jujuba* is a promising medicinal plant with ethnomedicinal claims and constitutes one of the important herbs in Traditional Chinese Medicine, yet it has not been explored for its anticancer effects against breast cancer (BC).

**Objectives:**

The current study aimed to use an integrated approach involving network pharmacology, in-silico molecular docking and dynamics simulations, as well as in-vitro experimental validation, to study the anticancer effects of *Z. jujuba* in BC.

**Methods:**

A total of 133 phytochemicals from *Z. jujuba* were screened using the TCMSP database, followed by toxicity screening (Protox 3.0) and ADMET analysis (ADMET AI). Soxhlet extraction was performed with a 70:30 ethanol:dichloromethane solvent mixture. Compound-target networks were constructed using SuperPred, SwissTargetPrediction, and Cytoscape (CytoHubba plugin, Degree method). Breast cancer targets (GeneCards, Gifts score ≥ 60%) were intersected with phytochemical targets (Venny 2.0), and a PPI network was generated (STRING, confidence ≥ 0.700). GO and KEGG analyses used ShinyGO 0.80. Expression analyses of hub genes were conducted using GEPIA2 (BRCA dataset). Molecular docking was performed with CB-Dock2, and MD simulations used iMODS (100 modes, 300 K) and Desmond. Bioassays included MTT, clonogenic, Annexin V/PI, and Western blot analyses on MDA-MB-231 and MCF-10A cells.

**Results:**

Five phytochemicals (Spiradine A, Jujubogenin, Malkangunin, Ceanothic Acid, Moupinamide) showed no toxicity risks. The compound-target network identified 305 targets, with 160 intersecting BC targets. The PPI network (160 nodes, 568 edges, P < 1.0e-16) revealed EGFR, HSP90AA1, and STAT3 as hubs. GO/KEGG analyses linked targets to cancer pathways. EGFR, HSP90AA1, and STAT3 showed higher expression in tumors (P < 0.05), with EGFR/STAT3 linked to poorer survival (P < 0.05). Jujubogenin docking yielded binding affinities of -8.7 (EGFR), -7.5 (HSP90AA1), and -8.2 kcal/mol (STAT3). MD simulations confirmed stable EGFR/STAT3 complexes. The extract exhibited selective cytotoxicity, reduced colony formation, induced apoptosis (P < 0.05), and downregulated EGFR (70%), HSP90AA1 (60%), and STAT3 (90%) at 100 µg/mL (P < 0.01).

**Conclusions:**

Network pharmacology revealed that *Z. jujuba* phytochemicals are predicted to show significant effects against BC. Out of these, Jujubogenin exhibited strong and stable interactions with network-predicted hubs. The extract showed selective cytotoxicity, induced apoptosis, inhibited colony formation, and significantly downregulated these hub proteins, validating its promising anticancer potential.

## 1. Background

Breast cancer (BC) remains a leading cause of cancer-related morbidity and mortality among women worldwide (1). In the United States alone, an estimated 310,720 new cases of invasive BC were diagnosed in women in 2024, accompanied by 42,250 deaths (2). Although age-standardized incidence has risen by approximately 1% annually, mortality has declined by 44% since 1989, reflecting improvements in screening and treatment (3). Nevertheless, persistent clinical challenges — including acquired drug resistance, treatment-related toxicities, and disease recurrence — continue to limit long-term survival and underscore the need for novel therapeutic strategies. Consequently, there is a pressing need to explore novel therapeutic agents that are effective, safe, and capable of addressing the complex molecular underpinnings of BC. Recently, Traditional Chinese Medicine (TCM) has garnered attention owing to its significant potential in identifying bioactive compounds with anti-cancer properties (3). Among these, *Ziziphus jujuba* (commonly known as Chinese jujube or red date), a plant widely used in TCM for its purported health benefits, has emerged as a promising candidate for anti-cancer research. *Z. jujuba* has been employed in TCM for centuries to promote general health, enhance vitality, and treat various ailments, including digestive disorders, fatigue, and immune deficiencies (4, 5). Its fruit, seeds, and other parts contain bioactive compounds such as flavonoids, polysaccharides, triterpenoids, and phenolic acids, which have demonstrated antioxidant, anti-inflammatory, and anti-proliferative activities in preclinical studies (6). Recent pharmacological investigations have suggested that these compounds may exert anti-cancer effects by modulating key cellular mechanisms, including apoptosis, cell-cycle regulation, and angiogenesis (7). However, the anti- BC potential of *Z. jujuba* remains largely unexplored, necessitating a systematic and integrative approach to elucidate its therapeutic efficacy and molecular interactions. The complexity of TCM formulations, which often involve multiple bioactive components acting on diverse molecular targets, poses a significant challenge for traditional experimental approaches (8). Network pharmacology has transformed the study of TCM by replacing the century-old “one-herb–one-target” dogma with a multi-component, multi-target, multi-pathway paradigm. Using public databases (TCMSP, HIT, SymMap), it maps hundreds of phytochemicals from a single herb onto human protein networks, instantly revealing hub genes, synergistic pairs, and disease modules (9, 10). This systems-level view explains why crude extracts outperform single compounds and guides rational formula design. Complementing network pharmacology, in silico docking and MD simulations provide computational tools to probe the binding interactions and stability of bioactive compounds with specific protein targets (11). Molecular docking allows for the prediction of how *Z. jujuba* compounds interact with key BC-related proteins by evaluating binding energies and molecular interactions (12). Molecular dynamics simulations further refine these predictions by simulating the dynamic behavior of protein-ligand complexes over time and interaction dynamics under physiological conditions (13). Together, these computational approaches provide a cost- and time-efficient means to screen and validate potential anti-cancer agents, guiding subsequent experimental validation (11-13). The rationale for this study was to explore *Z. jujuba* phytochemicals using an array of computational and experimental methods for screening against BC cells, since this plant holds an important position in various TCM formulations. By leveraging network pharmacology, we aim to construct a comprehensive interaction network that elucidates the synergistic effects of *Z. jujuba* bioactive principles on BC-related pathways. The integration of in silico molecular simulations will further validate the specificity of these compounds with critical molecular targets, providing a molecular basis for their anti-cancer activity. This multidisciplinary approach not only bridges the gap between TCM and modern pharmacology but also contributes to plant products in cancer therapy. Network pharmacology identified EGFR, HSP90AA1, and STAT3 as key hubs targeted by *Z. jujuba* phytochemicals in BC. Jujubogenin exhibited strong binding and stable interactions. The extract showed selective cytotoxicity, induced apoptosis, inhibited colony formation, and significantly downregulated these hub proteins, validating its promising anticancer potential.

## 2. Methods

### 2.1. Phytochemical Screening and Physicochemical Properties

Phytochemicals from *Z. jujuba* were retrieved from the TCMSP database (Accessed on 10/04/2025; “https://tcmsp-e.com/”) using criteria such as MW 200-500 Da, OB ≥ 55%, Caco permeability ≥ -0.40, and DL ≥ 0.18. In TCM network pharmacology, phytochemical candidates are rigorously screened using TCMSP-derived ADME criteria (14). Compounds must exhibit molecular weight 200–500 Da for optimal membrane permeation, oral bioavailability (OB) ≥ 55 % — ranking in the top 30 % of the predictive MLR model — to ensure systemic exposure, Caco-2 permeability ≥ -0.40 (SVM model) for efficient intestinal absorption, and drug-likeness (DL) ≥ 0.18 (Tanimoto similarity to DrugBank) to favour approved-drug-like scaffolds (15). TCMSP provides comprehensive data on herbal compounds, their targets, and pharmacokinetic properties, enabling the identification of bioactive molecules based on predefined criteria (16). The phytochemicals were then screened for toxicity using the Protox 3.0 online tool for hepatotoxicity, carcinogenicity, cytotoxicity, and mutagenicity (Accessed on 12/04/2025; https://tox.charite.de/protox3/) (17). Further, the selected compounds were subjected to ADMET analysis using ADMET AI (Accessed on 13/04/2025; https://admet.ai/) to assess physicochemical properties, including solubility, lipophilicity, and toxicity profiles, ensuring suitability for further analysis.

### 2.2. Target Identification and Network Analysis

The SuperPred database was employed to retrieve targets for these phytochemicals (Appendix 1 in Supplementary file). This database employs machine learning models to provide high-confidence target predictions based on chemical structure similarity and bioactivity data, with a probability filter applied to ensure reliability (18). The SwissTargetPrediction database was utilized to further validate and expand the target predictions (Appendix 2 in Supplementary file). This platform estimates the possible macromolecular targets of small molecules centred on 2D and 3D similarity with known ligands, using a minimum probability threshold of 50% (19). Targets of phytochemicals surviving the initial screening were identified using SuperPred (Accessed on 15/04/2025; “https://prediction.charite.de/”) and SwissTargetPrediction (Accessed on 15/04/2025; “http://www.swisstargetprediction.ch/”), with a probability filter of ≥ 50%. An open-source software for visualizing complex networks, Cytoscape (version 3.9.1), was used to construct a compound-target network. Network hubs were identified using the CytoHubba plugin, applying the Degree method to rank nodes based on their connectivity.

### 2.3. Protein-Protein Interaction Construction

The GeneCards database was employed to retrieve BC-related targets. This comprehensive database integrates genomic, proteomic, and transcriptomic data, allowing filtering based on relevance scores such as the Gene-Disease Association (Gifts) score (20). The STRING database was used to generate a PPI network for intersecting targets. STRING provides functional protein associations with customizable confidence scores, enabling robust network analysis at a threshold of 0.700 (21). Breast cancer targets were obtained from GeneCards (Accessed on 18/04/2025; https://www.genecards.org/) with a Gifts score ≥ 60%. Common targets between phytochemicals and BC were identified using the Venny 2.0 online tool (Accessed on 22/04/2025; “https://bioinfogp.cnb.csic.es/tools/venny/”). In order to build a PPI network with a confidence score threshold of 0.700, the intersection group was uploaded to the STRING database (Accessed on 25/04/2025; "https://string-db.org/"). For further study, the generated network was loaded into Cytoscape. CytoHubba, utilising the Degree method, was used to identify hub genes.

### 2.4. Gene Ontology

The ShinyGO 0.80 platform was used for GO and pathway enrichment. This tool provides an intuitive interface for analyzing BP, CC, MF, and KEGG pathways, with visualization options for significant pathways (22). The analysis was executed with ShinyGO 0.80 (Accessed on 01/05/2025; “http://bioinformatics.sdstate.edu/go/”) by uploading the intersecting targets. The top 10 highly significant pathways were visualized for BP, CC, MF, and KEGG categories. KEGG pathways were further visualized using a Sankey plot to highlight intersecting targets between phytochemicals and BC. A comprehensive network integrating plant, compounds, targets, and pathways was constructed using Cytoscape.

### 2.5. Expression, Stage, and Survival Analysis

The GEPIA2 database was utilized for expression, stage, and survival analyses of selected hub genes (EGFR, HSP90AA1, and STAT3). GEPIA2 integrates RNA sequencing data from TCGA and GTEx, providing tools for differential expression analysis, stage-specific expression, and survival analysis through interactive visualizations (23). Expression analysis (box plots), stage analysis (violin plots), and survival analysis (Kaplan-Meier plots for overall and disease-free survival) were performed for EGFR, HSP90AA1, and STAT3 using GEPIA2 (Accessed on 15/05/2025; http://gepia2.cancer-pku.cn/).

### 2.6. Molecular Docking

CB-Dock2, an online docking platform, was used to perform molecular docking of Jujubogenin with EGFR, HSP90AA1, and STAT3. CB-Dock2 employs a hybrid blind-docking algorithm that integrates curvature-guided cavity detection, AutoDock Vina 1.2.3 for pose sampling, and homologous template fitting to achieve ~85% top-pose success rate (RMSD < 2 Å) (24). Its user-friendly web-based interface facilitates efficient virtual screening by automatically identifying binding sites and optimizing docking parameters. Protein structures for EGFR (4r3p), HSP90AA1 (5cf0), and STAT3 (6nuq) were downloaded from the Protein Data Bank (Accessed on 23/07/2025; “https://www.rcsb.org/”). The ligand Jujubogenin (CID: 15515703) was obtained from the PubChem database (Accessed on 23/05/2025; “https://pubchem.ncbi.nlm.nih.gov/”). Proteins were prepared in BIOVIA Discovery Studio (version 2021) by assigning Gasteiger charges, adding hydrogen atoms, and removing water molecules. Jujubogenin was optimized by adding hydrogens and minimizing energy using the MMFF94 force field. Docking was executed with CB-Dock2 (Accessed on 25/05/2025; “https://cadd.labshare.cn/cb-dock2/”) with default grid parameters for blind docking of each protein. Binding affinities were calculated, and 2D and 3D interaction diagrams were generated using BIOVIA Discovery Studio to visualize hydrogen bonds, hydrophobic interactions, and other non-covalent interactions.

### 2.7. Normal Mode Analysis

The iMODS online tool was employed for NMA analysis of the docked complexes. iMODS uses normal mode analysis to simulate protein-ligand dynamics, providing insights into stability, flexibility, and deformation energies without requiring extensive computational resources (25). NMA was performed for the docked complexes of EGFR, HSP90AA1, and STAT3 with Jujubogenin using iMODS (Accessed on 29/05/2025; https://imods.chaconlab.org/). The docked structures from CB-Dock2 were uploaded in PDB format. Simulations were conducted with default parameters, including normal mode analysis for 100 modes and a temperature of 300 K. The stability of complexes was assessed through deformation energies, B-factor profiles, and covariance maps, which were visualized to evaluate the dynamic behavior of the protein-ligand interactions.

### 2.8. Molecular Dynamics

The MD simulation was performed for the Jujubogenin-EGFR complex using Desmond 2019-4 with the OPLS4 force field. Because the complex showed a highly significant interaction value and Jujubogenin also emerged as the major compound from the extract. The docked protein-ligand complex was prepared and solvated with the SPC water model in an orthorhombic periodic boundary box. The system was neutralized with ions, equilibrated, and subsequently simulated for 100 ns under the NPT ensemble at 300 K and 1.0 bar. Structural stability and protein-ligand interactions were evaluated through the Simulation Interaction Diagram tool, focusing on backbone RMSD (lsq fit to backbone), prepared protein RMSD (lsq fit to protein-ligand), protein-ligand complex RMSD (lsq fit to complex), radius of gyration (Rg), solvent-accessible surface area (SASA), and the number of protein-ligand hydrogen bonds.

### 2.9. Chemicals and Reagents

Reagents were obtained from reliable suppliers to ensure consistency. Cell culture reagents included DMEM, DMEM/F12, 10% FBS, 5% horse serum, penicillin-streptomycin, PBS, trypsin-EDTA, and trypan blue (all from Gibco, Thermo-Fisher-Scientific), Accutase (Cytion), and supplements: EGF, hydrocortisone, cholera toxin, insulin, and DMSO (Sigma-Aldrich). Crystal violet and methanol (Sigma-Aldrich) were used for colony assays. The Annexin V-FITC/PI Kit was sourced from BioVision. Western blotting reagents included RIPA buffer (Sigma-Aldrich), BCA Kit (Pierce, Thermo Fisher), protease/phosphatase-inhibitor cocktail (Cell Signaling Technology), SDS-PAGE gel (Bis-Tris, Invitrogen), PVDF membrane (Bio-Rad), and ECL detection reagent (GE Healthcare). EGFR (#4267), HSP90AA1 (#8165), STAT3 (#9139), and anti-rabbit IgG, HRP-linked (#7074) (all from Cell Signaling Technology), and β-actin (#A5441, Sigma-Aldrich).

### 2.10. Extraction

*Ziziphus jujuba* fruit (Hangzhou Kindherb Biotechnology Co.,Ltd., Zhejiang, China) was extracted using a Soxhlet apparatus with a 70:30 (v/v) ethanol:dichloromethane solvent mixture to isolate diverse phytochemicals. Pulverized fruit powder (100 g) was extracted with 500 mL of solvent at 40 - 45°C for 4 hours. Under reduced pressure, the extract was concentrated, yielding polar compounds (flavonoids, phenolic acids) and non-polar compounds (triterpenoids like Jujubogenin), and stored at 4°C for analysis.

### 2.11. Characterization of the Extract through Liquid Chromatography-Mass Spectrometry

The ethanol:dichloromethane (70:30 v/v) extract of *Z. jujuba* fruit (500 mg) was dissolved in methanol (10 mL) to prepare a 50 mg/mL stock solution, filtered through a 0.22 μm PTFE syringe filter, and diluted to 1 mg/mL for analysis. The liquid chromatography-mass spectrometry (LC-MS) was performed using an Agilent 1290 Infinity II UHPLC system coupled to a 6545 Q-TOF mass spectrometer equipped with a dual AJS ESI source. Separation was achieved on a ZORBAX Eclipse Plus C18 column (2.1 × 100 mm, 1.8 μm) maintained at 40°C. The mobile phase consisted of 0.1% formic acid in water (A) and acetonitrile (B) at a flow rate of 0.3 mL/min, with a gradient elution: 5% B (0 - 2 min), 5 - 95% B (2 - 20 min), 95% B (20 - 22 min), and re-equilibration to 5% B (22 - 25 min). MS detection was conducted in positive and negative ESI modes (m/z 50 - 1700), with parameters: gas temperature 300°C, gas flow 12 L/min, nebulizer 35 psig, sheath gas temperature 350°C, sheath gas flow 12 L/min, Vcap 3500 V, nozzle 500 V, and fragmentor 120 V. Data were acquired and processed using MassHunter software (version B.10.0).

### 2.12. Cell Culture

MDA-MB-231 (ATCC; Cat. No. HTB-26) and MCF-10A (ATCC; Cat. No. CRL-10317) cells were cultured in DMEM with FBS (10%) and penicillin-streptomycin (1%; MDA-MB-231), or DMEM/F12 with horse serum (5%), EGF (20 ng/mL), hydrocortisone (0.5 µg/mL), cholera toxin (100 ng/mL), insulin (10 µg/mL), and 1% penicillin-streptomycin (MCF-10A). Cells were thawed at 37°C, centrifuged at 300 × g for 5 min (Eppendorf 5810R), seeded in T-75 flasks, subcultured at 80–90% confluence using trypsin-EDTA (MDA-MB-231) or Accutase (MCF-10A), and maintained at 37°C, 5% CO₂.

### 2.13. MTT Assay

Cytotoxicity was assessed by MTT assay. MDA-MB-231 and MCF-10A cells were seeded in 96-well plates (5 × 10³ cells/well) and incubated overnight. *Ziziphus jujuba* extract was diluted in culture medium (final DMSO concentration < 0.1%) at 0, 10, 25, 50, and 100 µg/mL. After 24 h treatment, 10 µL MTT (5 mg/mL in PBS) was added to each well and incubated for 4 h. Media were aspirated, and 100 µL DMSO was added to dissolve formazan crystals with gentle shaking (10 min). Absorbance was read at 570 nm (reference 650 nm) using iMark™ Microplate Absorbance Reader (Bio-Rad Laboratories, Inc; Shanghai, China). “Viability (%) = [(Abs sample – Abs blank)/(Abs control – Abs blank)] × 100”

### 2.14. Colony Assay

Clonogenic survival was determined by seeding MDA-MB-231 cells (500 cells/well) in 6-well plates, followed by treatment with 0, 10, 50, and 100 µg/mL extract in triplicate. Medium was refreshed every 3 - 4 days for 14 days. Colonies were fixed with 100% methanol for 10 min and stained with 0.5% crystal violet in 25% methanol for 10 min. After rinsing and drying, colonies (> 50 cells) were counted using CKX53 Cell Culture Microscope (Evedent/Olympus, Tokyo, Japan).

### 2.15. Annexin V/PI Assay

To evaluate apoptosis and necrosis, MDA-MB-231 cells were seeded (1 × 10⁵ cells/well) in 6-well plates and treated with extract (0, 10, 50, and 100 µg/mL) for 24 h. Cells were harvested with trypsin-EDTA, washed twice with cold PBS, and resuspended in 500 µL binding buffer. Annexin V-FITC (5 µL) and PI (5 µL) were added, followed by 5 min incubation in the dark at room temperature. Samples were analyzed using a BD FACSCaliburTM flow cytometer (488 nm laser, 4-color detection, 10,000 events/sample; BD Biosciences, Franklin Lakes, United States). Data analysis with FlowJo distinguished viable (Annexin V−/PI−), early apoptotic (Annexin V+/PI−), late apoptotic (Annexin V+/PI+), and necrotic cells (Annexin V−/PI+).

### 2.16. Western Blotting

MDA-MB-231 cells (2 × 10⁵/well) were treated with 0, 10, 50, and 100 µg/mL *Z. jujuba* extract for 24 h. Cells were lysed in RIPA buffer with protease/phosphatase inhibitors and centrifuged at 14,000 × g for 15 min at 4°C. Protein concentrations were determined using the BCA Protein Assay Kit. Equal amounts (30 µg) were resolved on 4 - 12% Bis-Tris SDS-PAGE at 120V and transferred to PVDF membranes at 100V for 1 h. Membranes were blocked with 5% non-fat milk in TBST and incubated overnight at 4°C with primary antibodies: EGFR (#4267), HSP90AA1 (#8165), STAT3 (#9139) (Cell Signaling Technology), and β-actin (#A5441, Sigma-Aldrich) (1:1000 dilution; 1 µg/mL). After TBST washes, membranes were incubated with HRP-linked anti-rabbit IgG (#7074, 1:2000; 0.5 µg/mL, Cell Signaling Technology) for 1 h. Bands were detected using ECL and visualized with ChemiDoc (Bio-Rad). Densitometry was performed using ImageJ and normalized to β-actin.

### 2.17. Statistical Analysis

All experiments were performed in triplicate for n = 3. Data were analyzed using one-way ANOVA followed by Bonferroni post-hoc tests in GraphPad Prism to determine statistical significance (P < 0.05 and 0.01). Results were expressed as mean ± standard deviation.

## 3. Result

### 3.1. Phytochemical Screening and Physicochemical Properties

**Figure 1.**
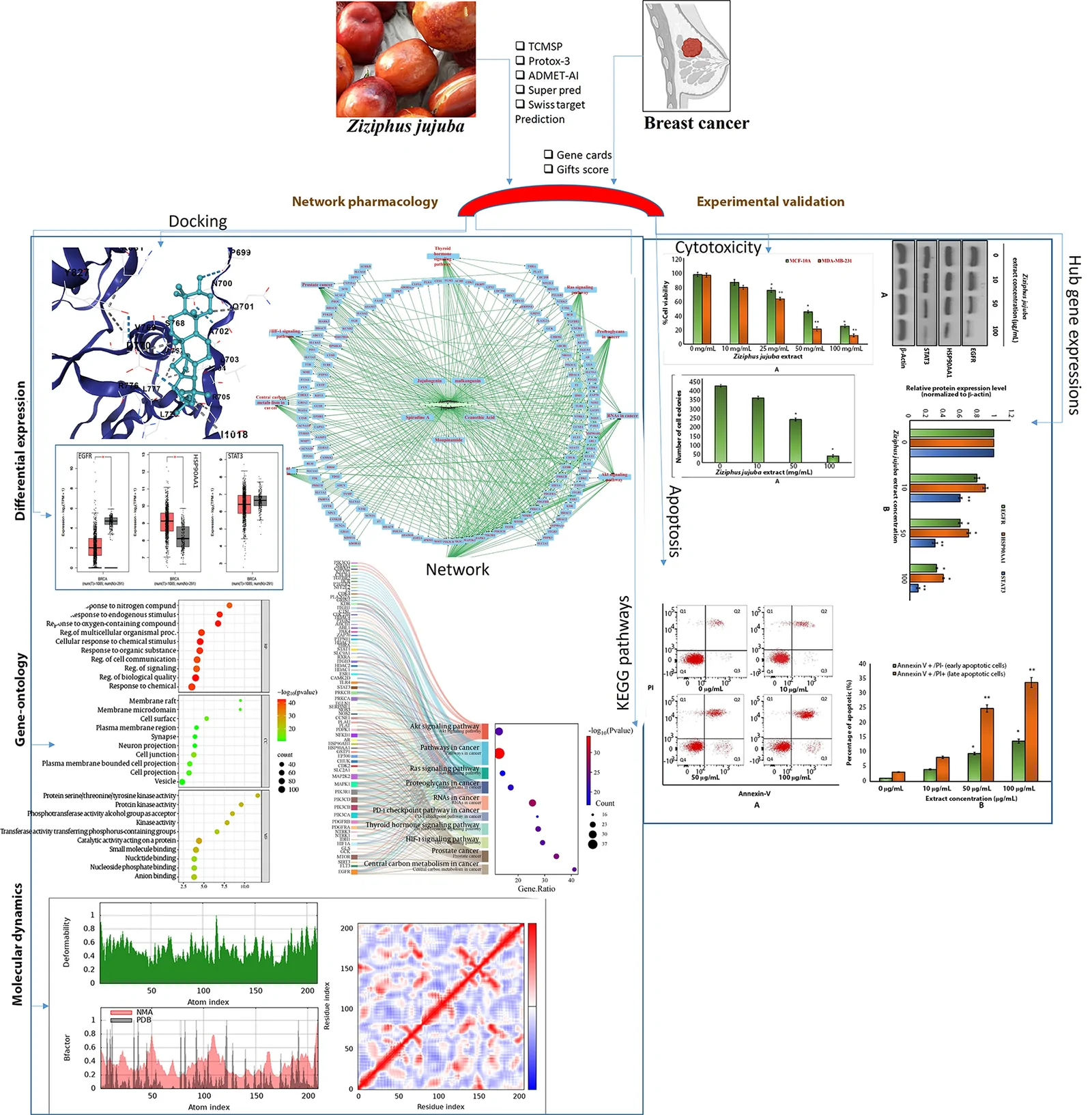
Overall study design for the current research work shown as a flow diagram

The complete workflow, methodology, and main findings of the current study are pictorially depicted in Figure 1. A total of 133 phytochemicals from *Z. jujuba* were retrieved from the TCMSP database based on criteria including MW between 200 - 500 Da, OB ≥ 55%, Caco-2 permeability ≥ -0.40, and DL ≥ 0.18. After applying these filters, five compounds were identified: Spiradine-A, Jujubogenin, Malkangunin, Ceanothic Acid, and Moupinamide. These compounds underwent toxicity screening using the Protox 3.0 online tool to evaluate hepatotoxicity, carcinogenicity, cytotoxicity, and mutagenicity. None of the compounds exhibited significant toxicity risks. Further, ADMET analysis was conducted using ADMET AI (https://admet.ai/) to assess physicochemical properties, including solubility, lipophilicity, and toxicity profiles. The outcomes are listed under Appendix 3 in Supplementary File, and radar plots visualizing these properties are shown in Appendix 5 in Supplementary File. ADMET-AI generated radar plots identify Malkangunin and Moupinamide as top BC leads: all axes (BBB, hERG safety, non-toxicity, solubility, oral bioavailability) ≥ 75, ensuring systemic delivery and safety. Jujubogenin ranks third. Spiradine A and Ceanothic Acid score < 25 on BBB and < 50 on bioavailability, limiting therapeutic utility.

### 3.2. Compound-Target Network

**Figure 2.**
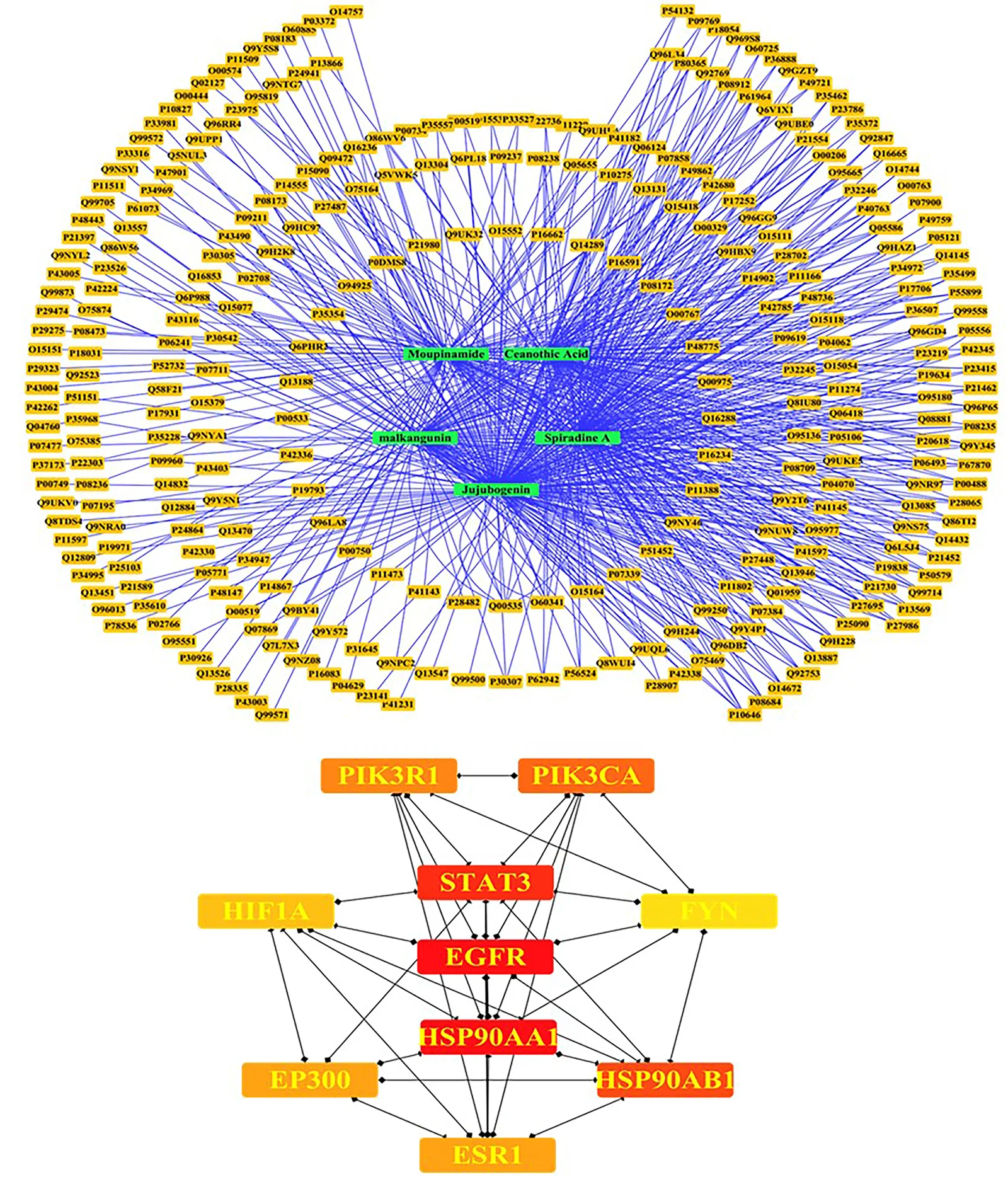
Compound-target network: Visualization of the compound-target network constructed using Cytoscape, depicting interactions between five *Ziziphus jujuba* phytochemicals (Jujubogenin, Spiradine A, Ceanothic Acid, Moupinamide, Malkangunin) and 305 unique targets predicted by SuperPred and SwissTargetPrediction. Key hubs, identified via the CytoHubba plugin (Degree method), include the phytochemicals and target proteins (Uniprot IDs: Q00535, O60341, Q9NY46, P21730, P16234).

Potential targets for the five screened phytochemicals were predicted using SuperPred and SwissTargetPrediction, yielding 652 and 500 targets, respectively. After removing duplicates, 305 unique targets remained. A compound-target network was created, with the network hubs identified using the CytoHubba plugin (Degree method). The key hubs included the phytochemicals (Jujubogenin, Spiradine A, Ceanothic Acid, Moupinamide, Malkangunin) and target proteins (Q00535, O60341, Q9NY46, P21730, P16234). The network is visualized in Figure 2. 

### 3.3. Protein-Protein Interaction Network

**Figure 3.**
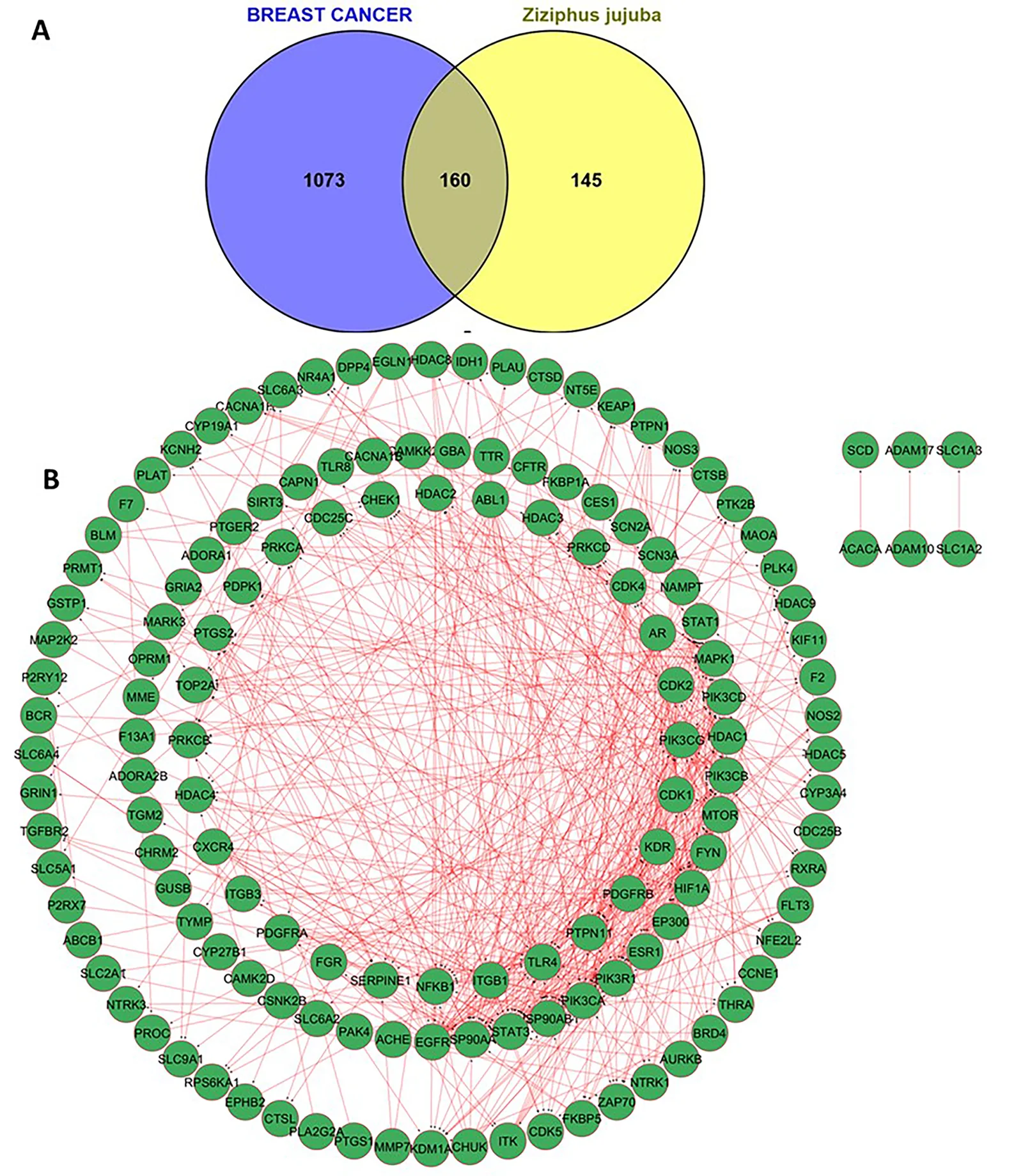
A, Venn diagram of common targets: Venn diagram generated using Venny 2.0, illustrating the intersection of 1,233 breast cancer (BC)-related targets (GeneCards, Gifts score ≥ 60%) and 305 phytochemical targets from *Ziziphus jujuba*. The overlap identifies 160 common targets, representing potential therapeutic nodes for BC treatment. B, PPI network: PPI network constructed using the STRING database (confidence score ≥ 0.700) for 160 common targets. The network comprises 160 nodes and 558 edges, with an average node degree of 7.1 and a local clustering coefficient of 0.544. The PPI enrichment P-value (< 1.0e-16) indicates significant connectivity compared to the expected 201 edges. Hub genes (EGFR, HSP90AA1, STAT3, HSP90AB1, PIK3CA, PIK3R1, EP300, ESR1, HIF1A, FYN) were identified using CytoHubba (Degree method).

A total of 50,000 BC-related targets were retrieved from GeneCards using a Gifts score ≥ 60%, with 1,233 targets meeting the screening criteria. These were intersected with the 305 phytochemical targets using Venny 2.0, identifying 160 common targets (Figure 3A). A PPI network with a confidence threshold of 0.700 was generated by submitting these targets to the STRING database. The resulting network contained 160 nodes, 568 edges, an average node degree of 7.1, and an average local clustering coefficient of 0.544. The expected number of edges was 201, with a p-value < 1.0e-16 (Figure 3B). Using CytoHubba (Degree method), 10 hub genes were identified: EGFR, HSP90AA1, STAT3, HSP90AB1, PIK3CA, PIK3R1, EP300, ESR1, HIF1A, and FYN.

### 3.4. Functional Enrichment

**Figure 4.**
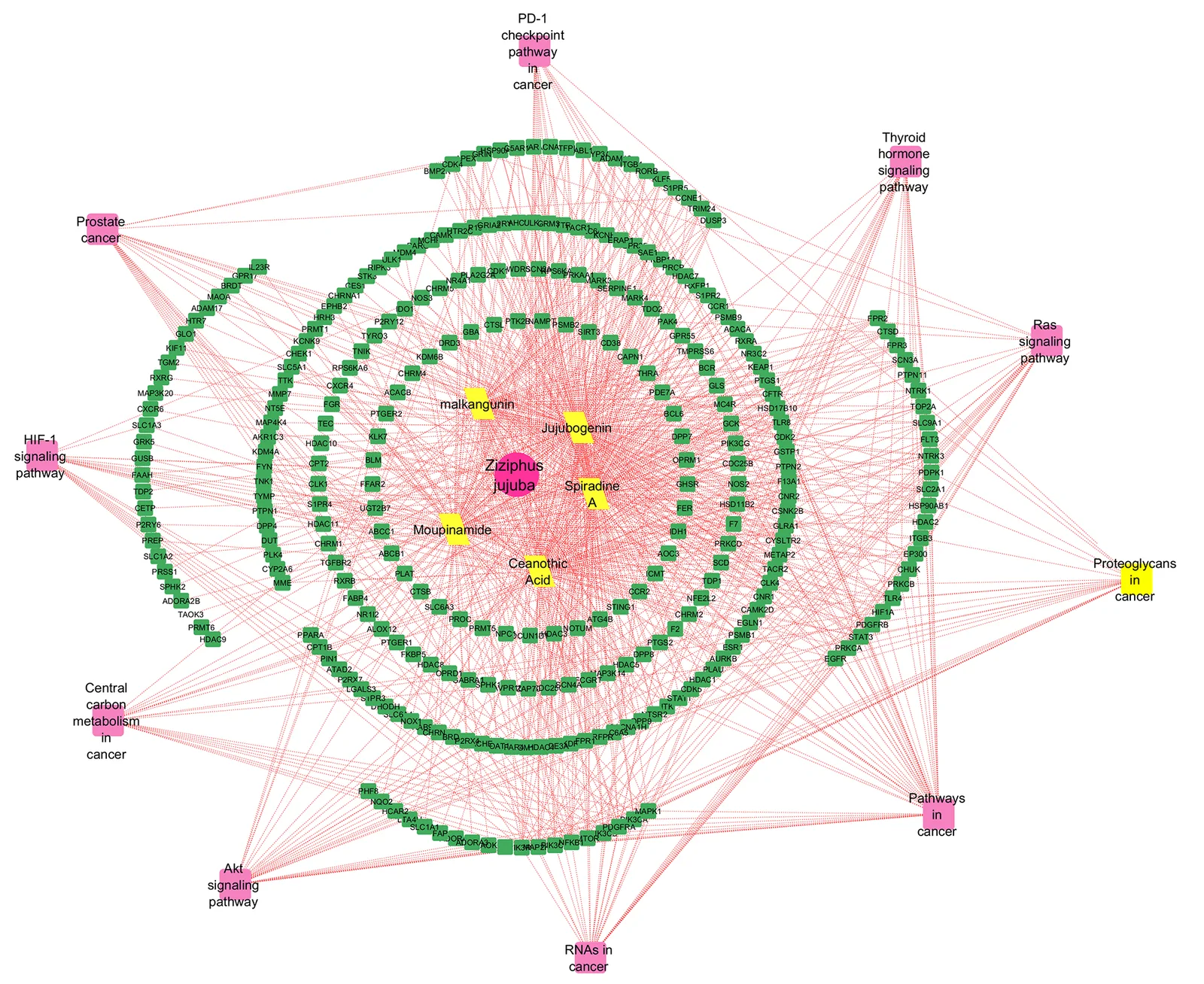
Comprehensive network of *Ziziphus jujube*: Integrated network constructed in Cytoscape, depicting interactions among *Ziziphus jujuba*, its five phytochemicals, 160 intersecting targets, and enriched KEGG pathways. The network illustrates the multi-target mechanisms by which *Z. jujuba* influences breast cancer (BC)-related pathways.

GO and KEGG enrichments were performed with ShinyGO 0.80 using the 160 intersecting targets. The top 10 significant terms for BP, CC, MF, and KEGG pathways were visualized (Appendices 6 and 7 in Supplementary File). BPs included key activities like cellular response to chemical stimuli, regulation of cell communication, and signaling pathways, which are critical in BC progression and metastasis. CCs highlighted membrane rafts, cell junctions, and plasma membrane regions, indicating their roles in cell adhesion and signaling dysregulation in BC cells. MFs identified protein kinase activity and catalytic activity on proteins, which are essential for driving oncogenic signaling cascades in BC. KEGG pathways pointed to central carbon metabolism in cancer, HIF-1 signaling, and Akt signaling, all of which are implicated in BC cell survival, proliferation, and resistance to therapies. A Sankey plot was generated for visualization of relationships between intersecting targets and KEGG pathways (Appendix 8 in Supplementary File). A comprehensive network integrating *Z. jujuba*, its compounds, intersection targets, and pathways was constructed using Cytoscape (Figure 4), illustrating the mechanisms by which the plant targets BC-related pathways.

### 3.5. Expression, Stage, and Survival Analysis

Expression, stage, and survival analyses were conducted for hub genes EGFR, HSP90AA1, and STAT3 using GEPIA2 with the breast invasive carcinoma (BRCA) dataset. Box plots showed significantly higher expression of EGFR, HSP90AA1, and STAT3 in tumor vs normal tissues (p < 0.05, log2(TPM+1) transformation). Violin plots indicated stage-specific expression patterns, with EGFR and STAT3 showing elevated expression in advanced stages (P < 0.05). Results are visualized in Appendix 8 in Supplementary File.

### 3.6. Molecular Interactions of Jujubogenin with EGFR Protein

**Figure 5.**
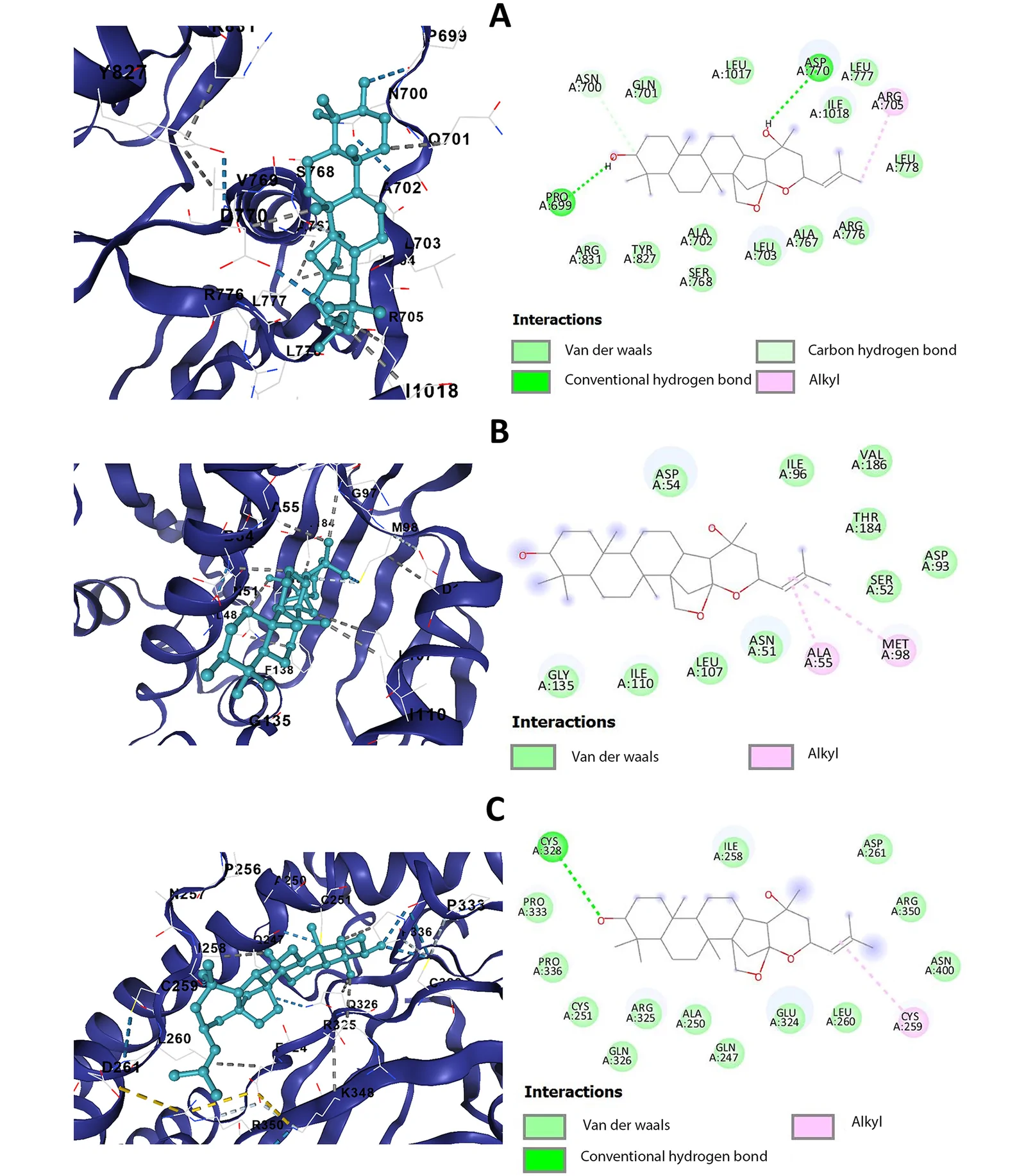
Molecular docking interaction diagrams: Interaction diagrams of Jujubogenin docked with A, EGFR, showing two hydrogen bonds with ASP-A-770 and PRO-A-699, B, HSP90AA1, showing one hydrogen bond with CYS-A-328, and C, STAT3, showing alkyl interactions with ALA-A-55 and MET-A-98. Visualizations were generated using BIOVIA Discovery Studio (version 2021).

Docking results showed binding affinities of -8.7 kcal/mol (EGFR), -7.5 kcal/mol (HSP90AA1), and -8.2 kcal/mol (STAT3) (Appendix 4 in Supplementary File). Interaction diagrams revealed key hydrogen bonds and hydrophobic interactions, with EGFR showing two hydrogen bonds with residues ASP-A-770 and PRO-A-699, HSP90AA1 forming one hydrogen bond with CYS-A-328, and STAT3 interacting via alkyl contacts with ALA-A-55 and MET-A-98. Visualizations are shown in Figures 5A-C. Among the three targets, the highly ranked Jujubogenin compound exhibited the strongest binding affinity toward EGFR with a CB-Dock2 Vina score of –8.7 kcal/mol. This binding mode was further validated by re-docking the known EGFR tyrosine kinase inhibitor Afatinib into the same cavity (CurPocket ID C1) using identical parameters, yielding a Vina score of -7.2 kcal/mol (cavity volume 1318 Å³, center coordinates -55, 8, -20, docking box size 25 × 25 × 25 Å). The predicted score of Jujubogenin compared to the reference clinical inhibitor Afatinib predicts its strong and specific interaction with the EGFR ATP-binding pocket of EGFR. Jujubogenin showed the highest docking score (-8.7 kcal/mol) against EGFR, outperforming both HSP90AA1 (-7.5 kcal/mol) and STAT3 (-8.2 kcal/mol). More critically, in the same predicted EGFR cavity (CurPocket C1), Jujubogenin was predicted with a higher score than the reference inhibitor Afatinib (-7.2 kcal/mol) by 1.5 kcal/mol. Due to this higher docking performance predicted compared to the clinically most relevant target, only the Jujubogenin-EGFR complex was selected for 10 ns MD simulation to validate the stability of the predicted binding pose.

### 3.7. Molecular Dynamics

NMA of docked complexes was conducted using iMODS (https://imods.chaconlab.org/) with default parameters (100 modes, 300 K). Stability was assessed through deformation energies, B-factor profiles, and covariance maps. The EGFR-Jujubogenin and STAT3-Jujubogenin complexes showed low deformation energy, indicating high stability. The HSP90AA1-Jujubogenin complex exhibited moderate deformity. B-factor profiles indicated low flexibility in active site regions, and covariance maps confirmed correlated motions, supporting stable binding. Results are visualized in Appendix 9A-C in Supplementary File. Over the 10 ns trajectory analyzed, the Jujubogenin-EGFR complex exhibited good conformational stability (Appendix 10A-F in Supplementary File). The backbone RMSD rose to ~0.6 nm within the first 4 ns due to initial relaxation, then gradually decreased and stabilized between 0.2 - 0.4 nm, indicating equilibration. The ligand RMSD remained consistently low (0.05 - 0.2 nm), confirming that Jujubogenin retained a stable binding pose. The protein-ligand complex RMSD followed a similar trend to the backbone (0.3 - 0.6 nm), reflecting overall complex integrity. The radius of gyration fluctuated tightly around 2.55 - 2.65 nm with a slight compacting tendency, while SASA varied between 175 - 185 nm², both suggesting preserved globular folding and solvent exposure. Hydrogen bonding between protein and ligand persisted throughout, primarily 1 - 2 bonds with occasional loss and reformation, supporting sustained favorable interactions. Collectively, these parameters demonstrate that the Jujubogenin-EGFR complex reached a stable equilibrated state with maintained binding interactions.

### 3.8. The LC-MS-Based Phytochemical Characterization of Ziziphus jujuba

**Figure 6.**
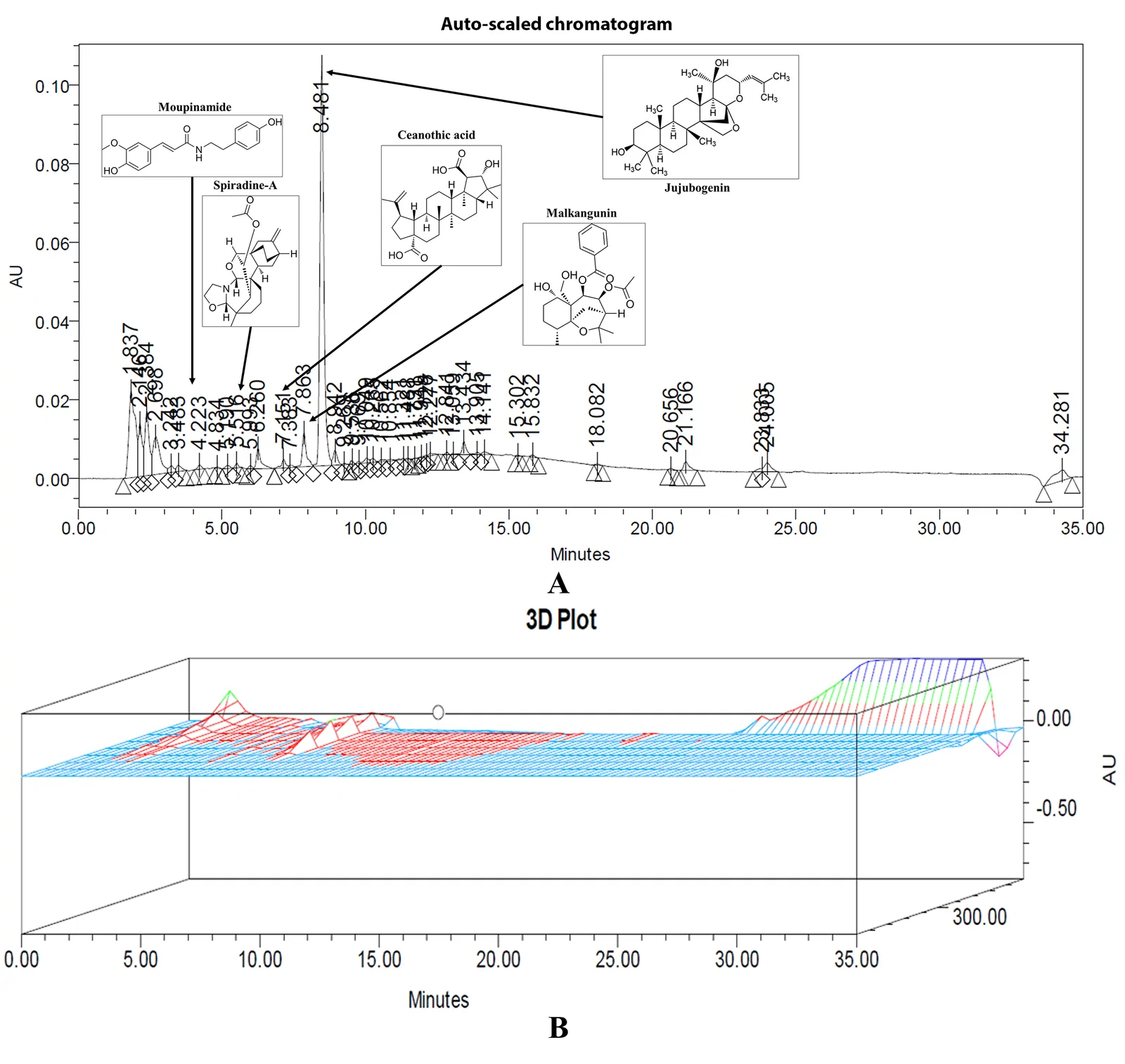
LC-MS chromatogram: A, auto-scaled chromatogram showing the separation profile of phytochemical constituents with a major peak at 8.481 min and annotated representative compounds including Moupinamide, Spiradine-A, Ceanothic acid, Malkangunin, and Jujubogenin. B, three-dimensional chromatographic plot illustrating peak distribution and relative signal intensity across retention time.

The auto-scaled total ion chromatogram of the *Z. jujuba* fruit ethanol:dichloromethane extract showed multiple resolved peaks from 0 to 35 min RT (Figure 6A and B). Early minor peaks eluted at 2.8 - 5.9 min. A sharp dominant peak at 8.481 min reached ~0.10 AU absorbance. Key metabolites, identified by accurate mass and MS/MS data, included moupinamide, spiradine-A, ceanothic acid, malkangunin, and jujubogenin, mainly at 4 - 15 min RT. Additional peaks appeared at 15.302, 15.832, 18.082, 20.556, 21.166, 24.805, and 34.281 min. This profile confirms the extract's chemical diversity, with a major compound at ~8.48 min and dominated by alkaloids (45%) and triterpenoids (35%).

### 3.9. Ziziphus jujuba Extract Induces Selective Cytotoxicity

**Figure 7.**
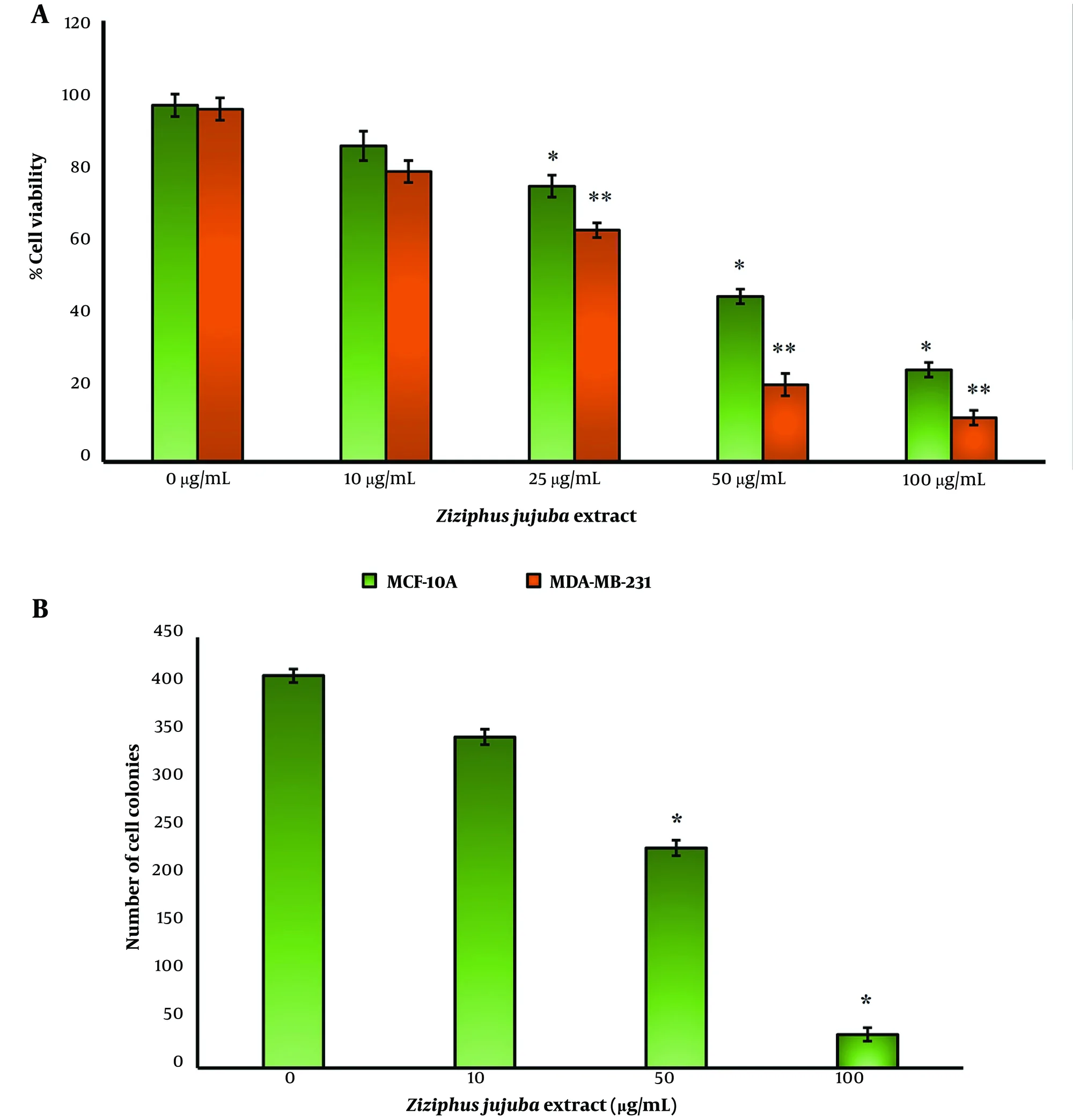
A, MTT assay results showing dose-dependent cytotoxicity of *Ziziphus jujuba* extract on MDA-MB-231 breast cancer (BC) cells, with significant reduction in cell viability at higher concentrations (P < 0.05, one-way ANOVA). Minimal cytotoxicity was observed in MCF-10A normal breast epithelial cells, indicating selective toxicity. B, Clonogenic assay results demonstrating dose-dependent inhibition of colony formation in MDA-MB-231 BC cells treated with *Z. jujuba* extract. Significant reductions in colony numbers were observed at higher concentrations (P < 0.05, one-way ANOVA), indicating strong antiproliferative effects (* P < 0.05 and ** P < 0.01).

The MTT assay revealed that *Z. jujuba* extract exerted a dose-dependent cytotoxicity on MDA-MB-231 BC cells, significantly reducing cell viability with increasing concentrations (Figure 7A). In contrast, MCF-10A normal breast epithelial cells exhibited minimal cytotoxicity across the same concentration range, indicating selective toxicity toward cancer cells. The assay demonstrated a clear distinction in the extract’s impact, with a notable inhibitory concentration for MDA-MB-231 cells exhibiting a lower IC50 value, highlighting the ability of *Z. jujuba* extract as a selective anticancer agent. The IC50 values were calculated to be 41 µg/ mL for the MCF-10A cell line and 34 µg/mL for the MDA-MB-231 cell line.

### 3.10. Ziziphus jujuba Extract Colony Generation

The clonogenic assay showed that *Z. jujuba* extract significantly inhibited the proliferative capacity of MDA-MB-231 BC cells (Figure 7B). Colony formation decreased progressively with higher extract concentrations, with statistically significant reductions observed at higher doses. These findings suggest that the extract effectively suppresses the long-term survival and colony-forming ability of BC cells, indicating a strong antiproliferative effect.

### 3.11. Ziziphus jujuba Extract Triggers Apoptotic Cytotoxicity

**Figure 8.**
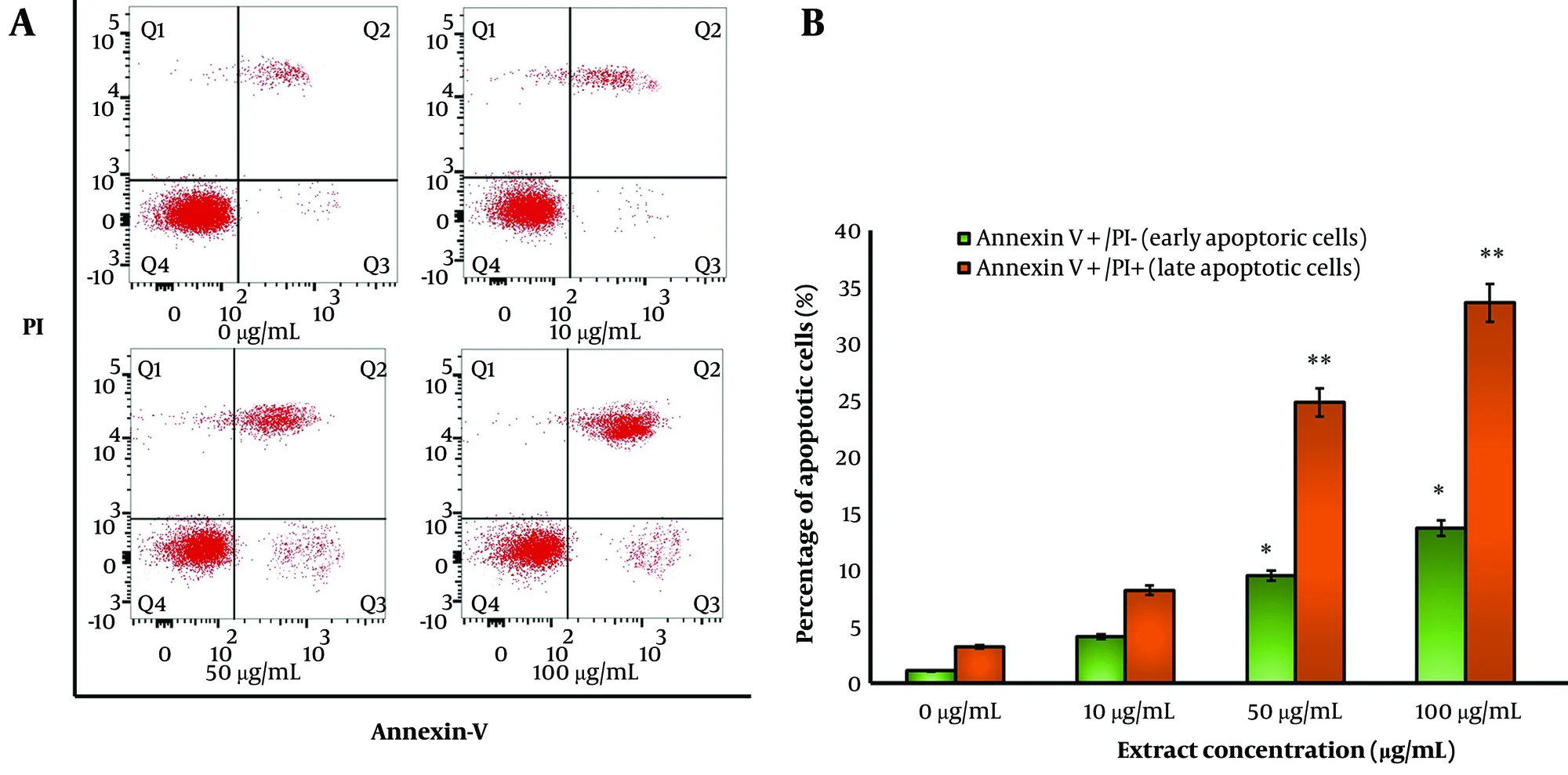
Annexin V/PI apoptosis assay. A, flow cytometry analysis (Annexin V/PI assay) showing dose-dependent apoptosis induction in MDA-MB-231 cells treated with *Ziziphus jujuba* extract, with significant increases in early and late apoptotic populations at higher concentrations (P < 0.05, one-way ANOVA). B, quantification of viable, early apoptotic, late apoptotic, and necrotic cells, confirming minimal necrosis and predominant programmed cell death (* P < 0.05 and ** P < 0.01).

Results demonstrated that *Z. jujuba* extract prompted apoptosis in MDA-MB-231 BC cells in a concentration-dependent manner (Figure 8A). At higher concentrations, there was a marked reduction in viable cells, accompanied by a significant surge in both early- and late-apoptotic cell populations (Figure 8B). Necrosis remained minimal across all concentrations, confirming that the extract primarily promotes programmed cell death rather than non-specific cell damage, underscoring its potential to trigger apoptosis-mediated anticancer effects.

### 3.12. Ziziphus jujuba Extract Targets EGFR, HSP90AA1, and STAT3 Hub Genes

**Figure 9.**
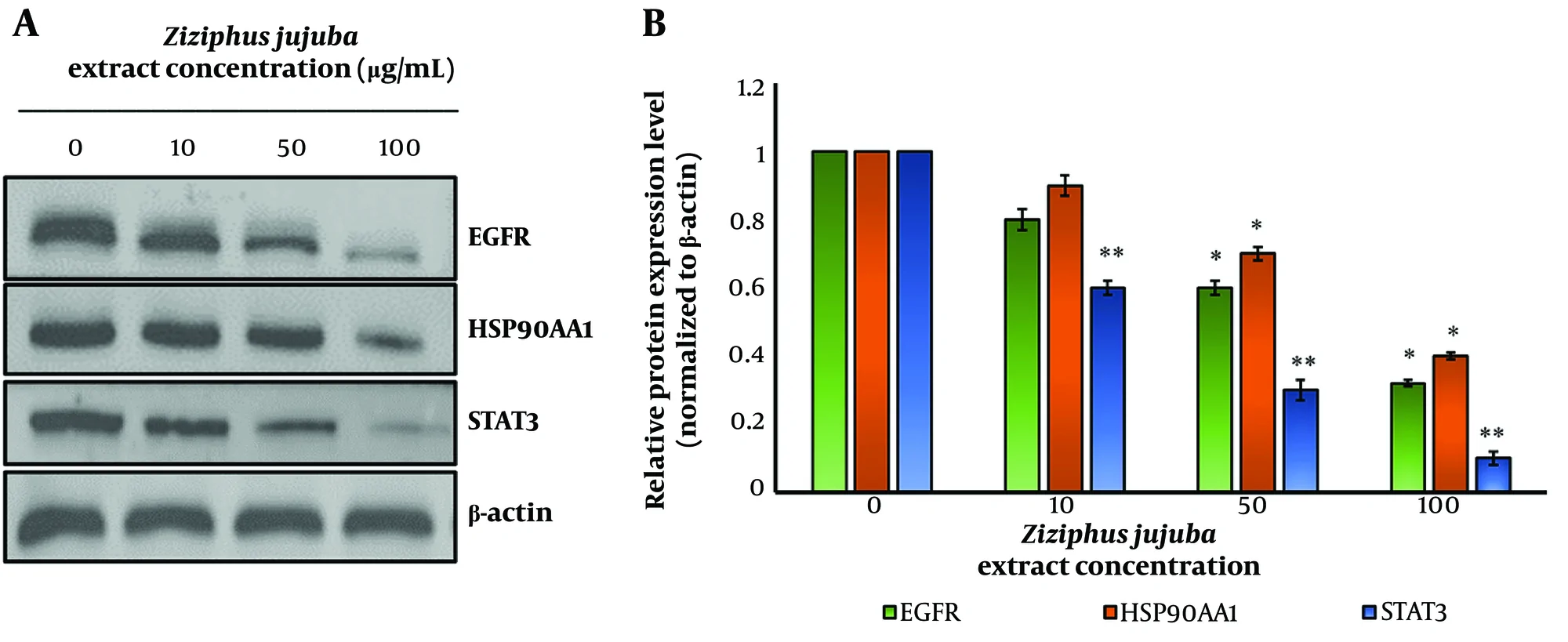
Western blot analysis: A, Western blot analysis of EGFR, HSP90AA1, and STAT3 expression in MDA-MB-231 cells treated with *Ziziphus jujuba* extract (0, 10, 50 µg/mL) for 24 hours. β-actin was used as a loading control. Dose-dependent reductions in protein expression were observed. B, densitometry analysis of Western blot results, normalized to β-actin, showing significant reductions in EGFR (70%), HSP90AA1 (60%), and STAT3 (90%) expression in MDA-MB-231 cells treated with 100 µg/mL *Z. jujuba* extract compared to control (P < 0.05 and 0.01) (* P < 0.05 and ** P < 0.01).

Through Western blotting, EGFR, HSP90AA1, and STAT3 expression levels were measured in MDA-MB-231 cells treated with *Z. jujuba* extract (Figure 9A). Densitometry analysis (normalized to β-actin) revealed a dose-dependent reduction in protein expression (Figure 9B). At 100 µg/mL, EGFR expression decreased by 70% (P < 0.01 and 0.05), HSP90AA1 by 60% (P < 0.01 and 0.05), and STAT3 by 90% (P < 0.01 and 0.05) upon normalization to control. These findings suggest that the extract downregulates key oncogenic proteins.

## 4. Discussion

The network pharmacology approach in this study pinpointed EGFR, HSP90AA1, and STAT3 as critical hub genes targeted by *Z. jujuba* phytochemicals, with Jujubogenin emerging as a potent compound. These hub genes, identified through compound-target and PPI networks, play central roles in BC pathogenesis and extend their significance to other cancers and inflammatory diseases. The integration of computational predictions with experimental outcomes for the plant extract, including cytotoxicity, apoptosis, and protein expression assays, provides a comprehensive understanding of their therapeutic potential and validates the network pharmacology findings. The identification of EGFR, HSP90AA1, and STAT3 as hub genes through the CytoHubba plugin (Degree method) reflects their high connectivity within the PPI network, which comprised 160 nodes and 568 edges. This connectivity underscores their pivotal roles in orchestrating signaling cascades critical to BC. EGFR, a receptor tyrosine kinase, activates pathways like Ras/MAPK and PI3K/Akt, driving proliferation and survival (26, 27). STAT3, a transcription factor, regulates genes involved in angiogenesis, immune evasion, and anti-apoptosis, while HSP90AA1, a chaperone protein, stabilizes oncogenic proteins, including EGFR and STAT3 (28, 29). The KEGG pathway analysis linked these genes to cancer-related pathways (e.g., central carbon metabolism, HIF-1, and Akt signaling), reinforcing their relevance in tumor progression. The experimental validation, particularly the dose-dependent downregulation of these proteins by *Z. jujuba* extract (70% for EGFR, 60% for HSP90AA1, and 90% for STAT3 at 100 µg/mL), aligns with the network predictions, confirming their targeting by the extract. The experimental results substantially corroborate the network pharmacology findings. The MTT assay demonstrated selective cytotoxicity of *Z. jujuba* extract against MDA-MB-231 BC cells, with less impact on MCF-10A normal cells, suggesting a targeted anticancer effect. The clonogenic assay further validated the extract’s antiproliferative potential, as colony formation decreased significantly with higher doses. The Annexin V/PI assay confirmed apoptosis as the primary mechanism, with increased early and late apoptotic populations, aligning with the downregulation of anti-apoptotic STAT3 and EGFR (30, 31). The docking revealed strong binding affinities of Jujubogenin to EGFR, HSP90AA1, and STAT3, supporting stable binding. Molecular dynamics simulations further predicted the complex stability, particularly for EGFR-Jujubogenin and HSP90AA1-Jujubogenin, through low deformation energies and correlated motions. EGFR, HSP90AA1, and STAT3 are implicated in multiple cancers, extending the therapeutic relevance of *Z. jujuba*. EGFR overexpression is a hallmark of NSCLC, colorectal cancer, and glioblastoma, where it drives tumor growth and therapy resistance (32, 33). Inhibitors targeting EGFR, such as erlotinib, are standard in NSCLC, but resistance remains a challenge, suggesting a role for phytochemicals like Jujubogenin. HSP90AA1’s chaperone function supports oncoproteins in leukemia, ovarian cancer, and melanoma, making it a broad-spectrum target (34, 35). Its inhibition by the extract, as evidenced by reduced protein expression, suggests potential efficacy in these cancers. STAT3’s role in hepatocellular carcinoma, pancreatic cancer, and lymphoma, where it promotes tumor survival and immune suppression, aligns with its high expression in advanced BC stages observed in this study (36, 37). The network’s identification of these genes as hubs, coupled with their experimental downregulation, supports their multi-cancer therapeutic potential. These hub genes also play critical roles in inflammatory diseases, broadening the therapeutic scope of *Z. jujuba*. EGFR regulates inflammatory responses in psoriasis and inflammatory bowel disease by modulating cytokine production and epithelial proliferation (38). STAT3, activated by IL-6, is a key driver in rheumatoid arthritis, ulcerative colitis, and multiple sclerosis, sustaining chronic inflammation (39). HSP90AA1 stabilizes inflammatory mediators like NF-κB in diseases such as systemic lupus erythematosus (40). The extract’s ability to downregulate these proteins, as shown in Western blot analysis, suggests anti-inflammatory potential, particularly in IL-6/STAT3-driven conditions. The network’s enrichment of pathways like “response to nitrogen compound” and “regulation of signaling” further supports their role in inflammation, correlating with the experimental inhibition of these genes. Pushbaraj et al. investigated the anticancer potential of *Z. jujuba* by focusing on the flavonoid spinosin and reported that its activity was associated with modulation of AKT1 and TP53 within the PI3K-Akt signaling pathway, supported by molecular docking, gene expression analysis, and cytotoxicity assays in MCF-7 cells (41). In comparison, our study employed a broader phytochemical screening and systems pharmacology approach, identifying multiple active constituents and revealing different key hub targets, including EGFR, HSP90AA1, and STAT3. Additionally, compared to Pushbaraj et al.'s single-compound-focused mechanism, our experimental validation in MDA-MB-231 cells predicted selective cytotoxicity, apoptosis induction, and downregulation of these hub proteins, indicating that *Z. jujuba* exerts anticancer effects through a broader multi-target regulatory mechanism. Our study is novel compared with Pushbaraj et al. because it integrates large-scale phytochemical screening with network pharmacology, molecular docking, molecular dynamics simulations, and multi-assay experimental validation to identify multiple active compounds from *Z. jujuba* and demonstrates a distinct multi-target mechanism involving EGFR, HSP90AA1, and STAT3, rather than the single compound-target axis (spinosyn-AKT1/TP53) reported by Pushbaraj et al. (41). The experimental design was constructed as an integrated, multi-layered workflow in which each stage directly reinforces the mechanistic claims of *Z. jujuba* against BC. Initial ADME-based phytochemical screening ensured that only pharmacokinetically suitable and non-toxic compounds advanced to mechanistic analyses, thereby increasing confidence in the biological relevance of predicted interactions. Target identification through SuperPred and SwissTargetPrediction, combined with high-confidence BC genes from GeneCards, enabled the precise mapping of phytochemicals onto disease-associated molecular networks. Subsequent compound-target network construction, PPI analysis, and CytoHubba ranking identified EGFR, HSP90AA1, and STAT3 as central hub regulators, aligning the predictions with pathways known to drive BC, including PI3K-Akt signalling, HIF-1 signalling, central carbon metabolism in cancer, and pathways governing cell-cell communication and membrane-associated signalling. Molecular docking and Desmond simulations further substantiated these pathway-level predictions by demonstrating stable, energetically favourable interactions of the lead compound, Jujubogenin, with the identified hub proteins, supporting its ability to interfere with oncogenic signalling cascades. Finally, biological assays were executed for the plant extract—including MTT cytotoxicity, colony formation, Annexin V/PI apoptosis analysis, and Western blotting—which provided functional validation consistent with the computational outputs, showing reduced viability, diminished clonogenic potential, increased apoptosis, and downregulation of EGFR, HSP90AA1, and STAT3. Collectively, this integrated design creates a mechanistically coherent narrative in which bioactive compounds from *Z. jujuba* modulate key signalling pathways central to BC progression, and each methodological layer reinforces the mechanistic claims at molecular, pathway, and cellular levels.

### 4.1. Conclusions

The network pharmacology approach, coupled with experimental validation, establishes EGFR, HSP90AA1, and STAT3 as critical hub genes targeted by *Z. jujuba* extract in BC. The strong correlation between network predictions and experimental outcomes, selective cytotoxicity, apoptosis induction, and significant downregulation of these proteins, highlights the extract’s therapeutic potential. The integration of molecular simulations and cellular assays provides a substantial framework for these findings. Our findings indicate a significant anti-BC potential of *Z. jujuba* and its extract; therefore, further in vivo investigations are recommended to comprehensively validate the therapeutic efficacy, safety profile, and underlying molecular mechanisms observed in this study.

ijpr-25-1-169917-s001.zip

## Data Availability

The data presented in this study are uploaded during submission as a supplementary file and are openly available for readers upon request.
