# Supplementary material for: Anticancer Effects of Ziziphus jujuba in Human Breast Carcinoma: An Integrated Study of Network Pharmacology, Molecular Docking and Dynamics Simulations and in-vitro Experimental Validation
Source: Iran J Pharm Res. 2026 Apr 20;25(1):e169917. doi: 10.5812/ijpr-169917 (PMC13558866; doi:10.5812/ijpr-169917)
Supplement: Supplementary file 1 [file ijpr-25-1-169917-s001.zip › Appendices 5-10.pdf]

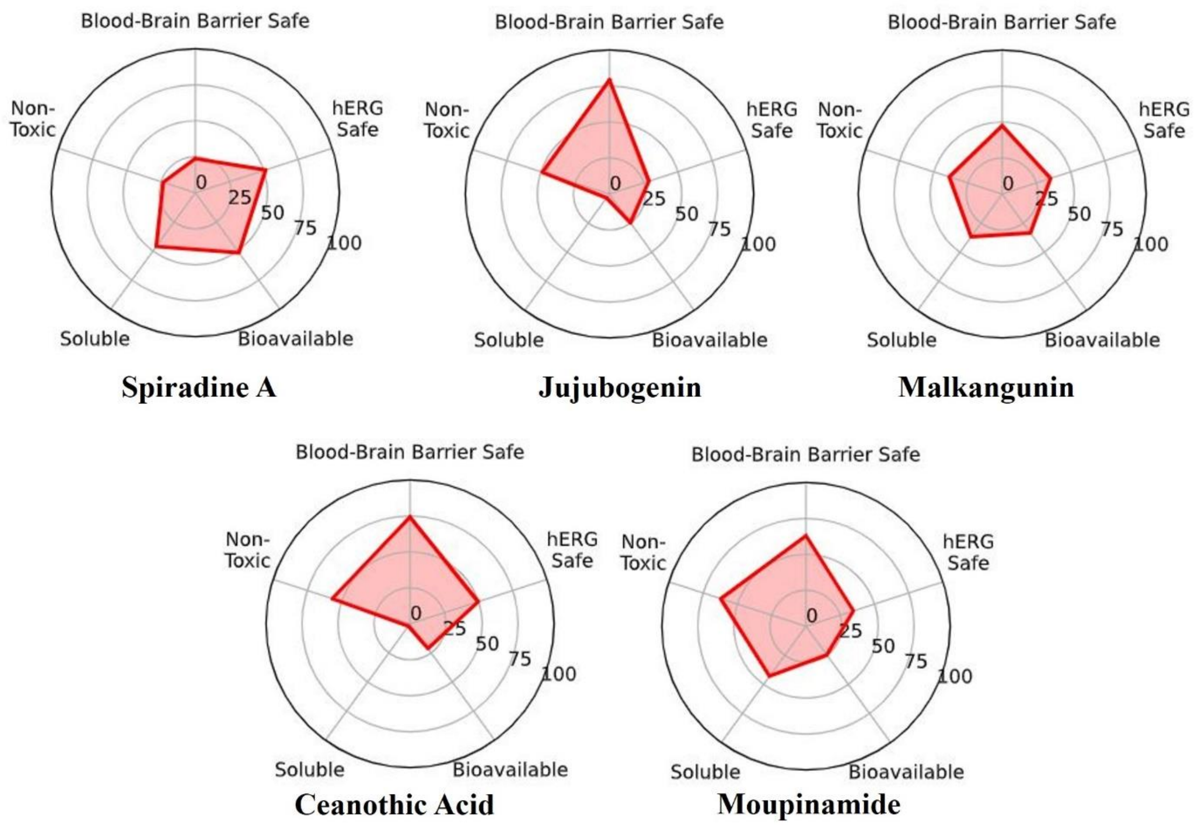

Appendix 5. Radar Plots of Physicochemical Properties: Radar plots illustrating the physicochemical properties of five *Ziziphus jujuba* phytochemicals (Spiradine A, Jujubogenin, Malkangunin, Ceanothic Acid, and Moupinamide) based on ADMET AI analysis. Parameters include oral bioavailability, toxicity, hERG safety, solubility and blood-brain barrier penetration. Each plot visualizes the relative values of these properties for individual compounds.

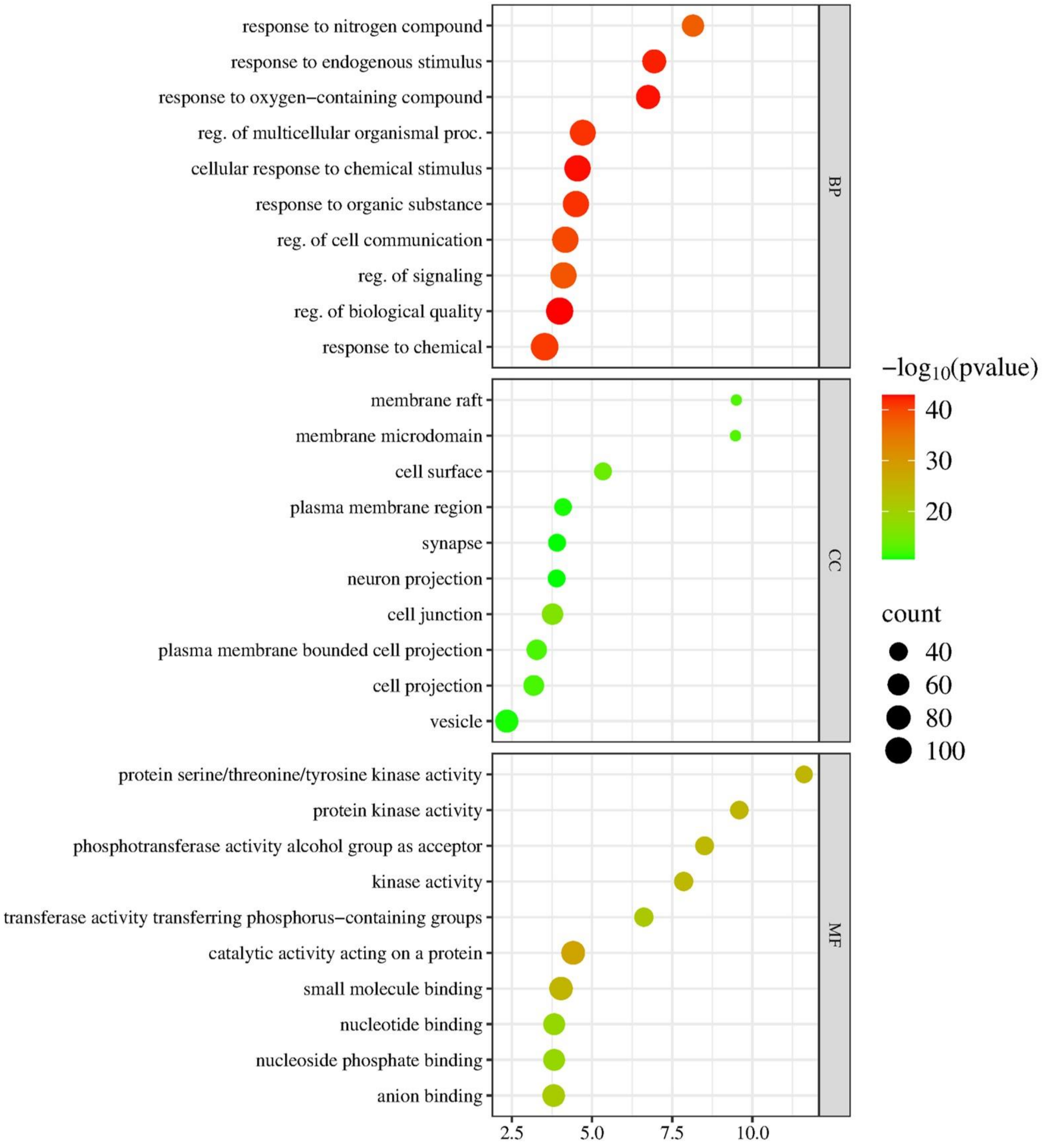

Appendix 6. GO Enrichment: Bar plots depicting the top 10 significant GO terms for 160 intersecting targets analyzed using ShinyGO 0.80. Categories include (**A**) Biological Process (e.g., response to nitrogen compound, $p < 0.05$), (**B**) Cellular Component (e.g., membrane raft, $p < 0.05$), and (**C**) Molecular Function (e.g., protein kinase activity, $p < 0.05$), illustrating the functional roles of *Ziziphus jujuba* targets in breast cancer-related processes.

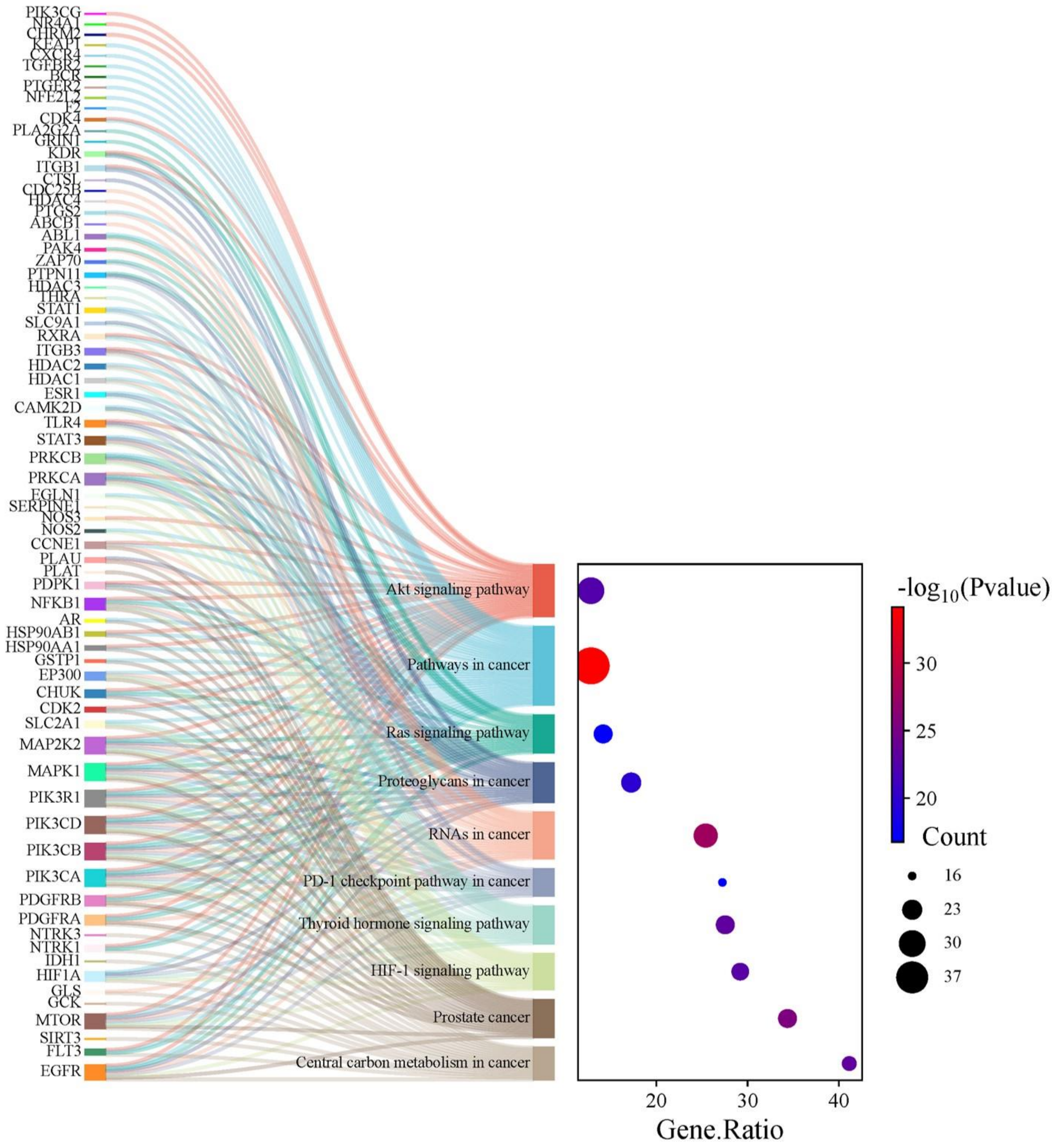

Appendix 7. KEGG Pathway Enrichment and Sankey Plot: Visualization of KEGG pathway enrichment analysis for 160 intersecting targets using ShinyGO 0.80, with a Sankey plot illustrating relationships between targets and the top 10 pathways (e.g., central carbon metabolism in cancer, HIF-1 signaling, $p < 0.05$). The plot highlights the mechanistic links between *Ziziphus jujuba* phytochemicals and breast cancer pathways.

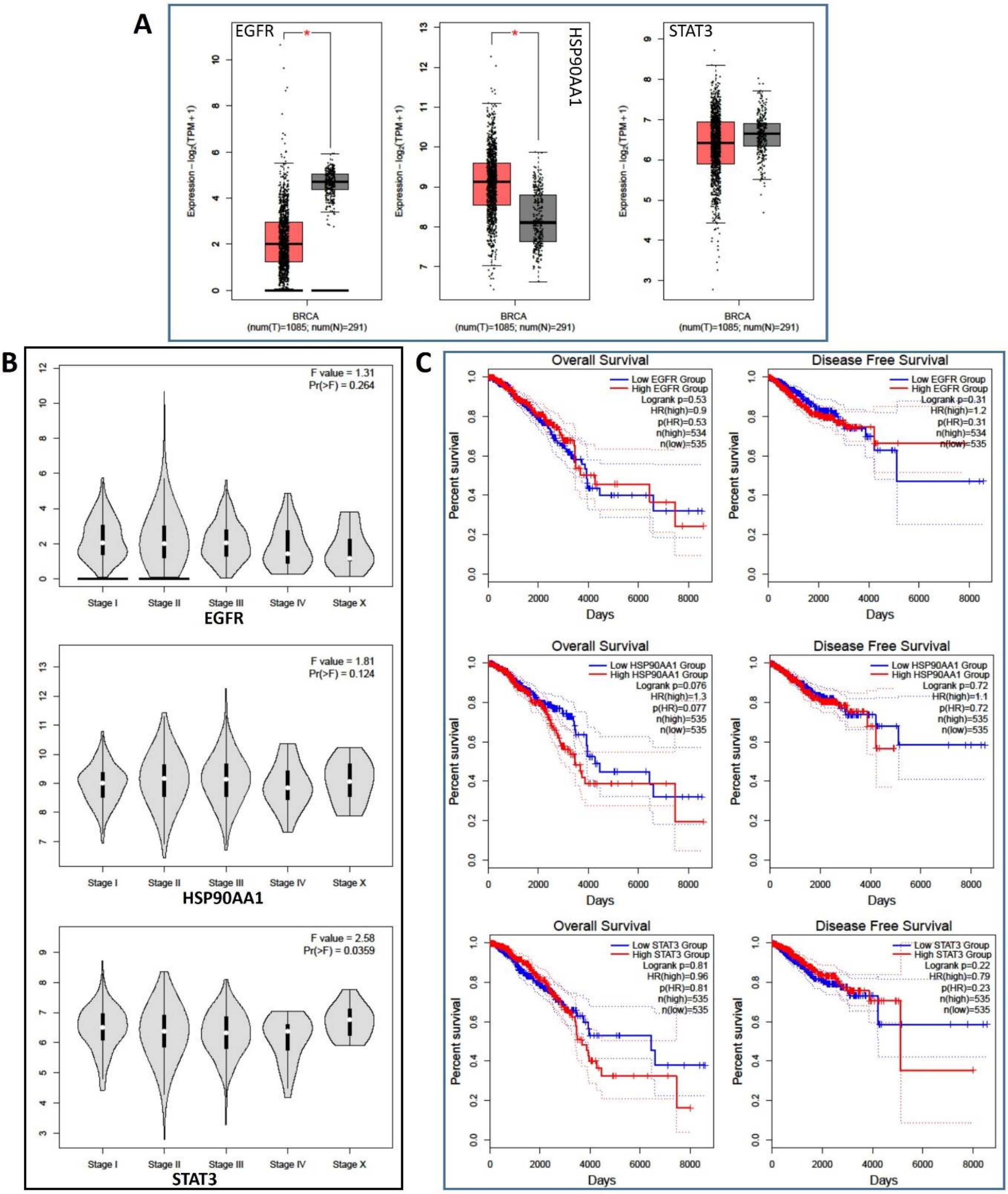

Appendix 8. Expression, Stage, and Survival Analysis: (**A**) Box plots showing significantly higher expression of EGFR, HSP90AA1, and STAT3 in breast invasive carcinoma (BRCA) tumor tissues compared to normal tissues ($p < 0.05$, log2(TPM+1) transformation, GEPIA2). (**B**) Violin plots indicating stage-specific expression patterns, with EGFR and STAT3 showing elevated expression in advanced stages ($p < 0.05$). (**C**) Kaplan-Meier plots demonstrating poorer overall and disease-free survival with high EGFR and STAT3 expression ($p < 0.05$), and moderate correlation for HSP90AA1.

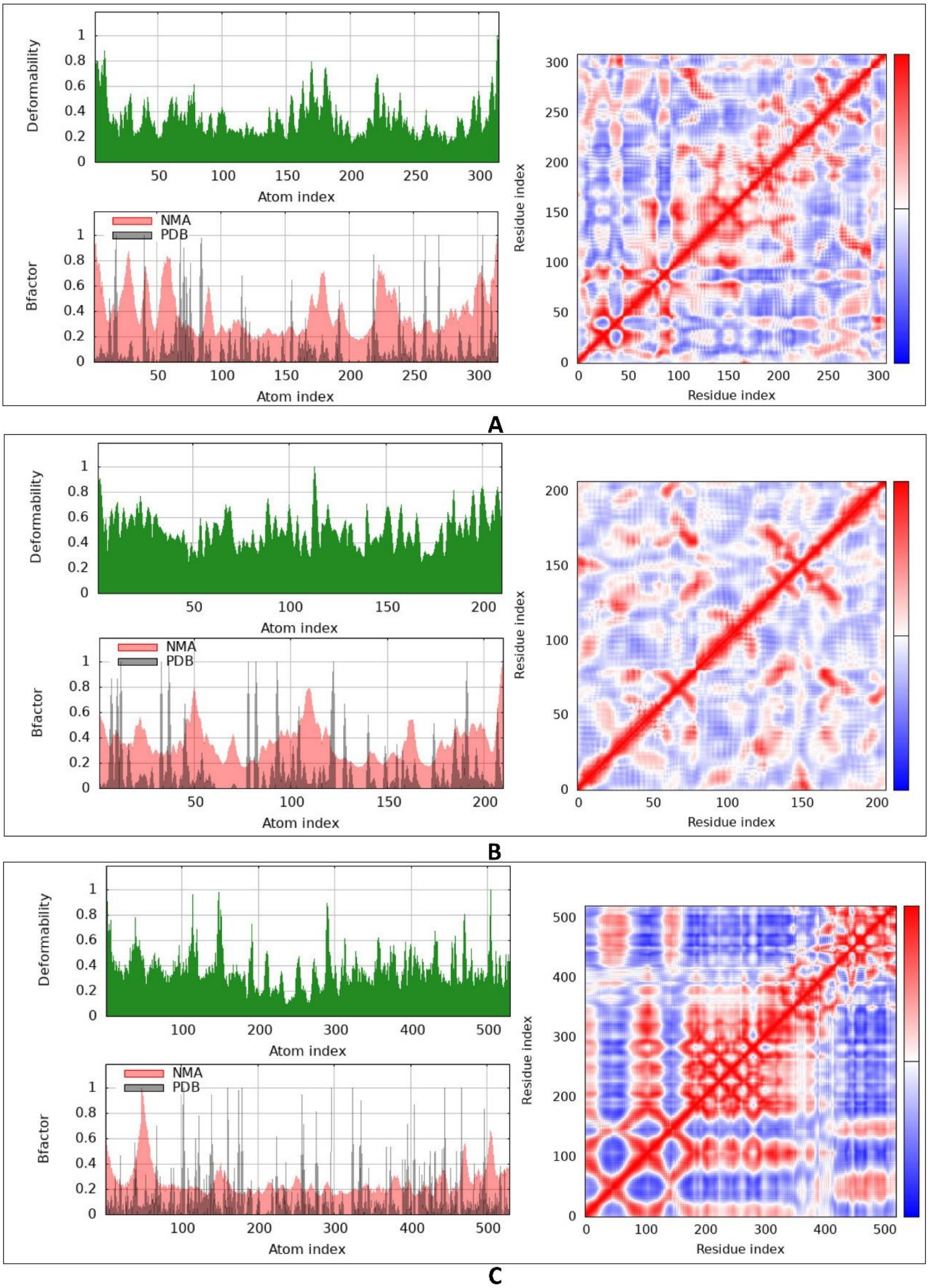

Appendix 9. Normal Mode Analysis: Normal mode analysis results for Jujubogenin complexes with (**A**) EGFR, (**B**) HSP90AA1, and (**C**) STAT3, conducted using iMODS (100 modes, 300 K). Visualizations include deformation energy profiles, B-factor profiles indicating low flexibility in active sites, and covariance maps showing correlated motions. EGFR and STAT3 complexes exhibit low deformation energy, while HSP90AA1 shows moderate deformity.

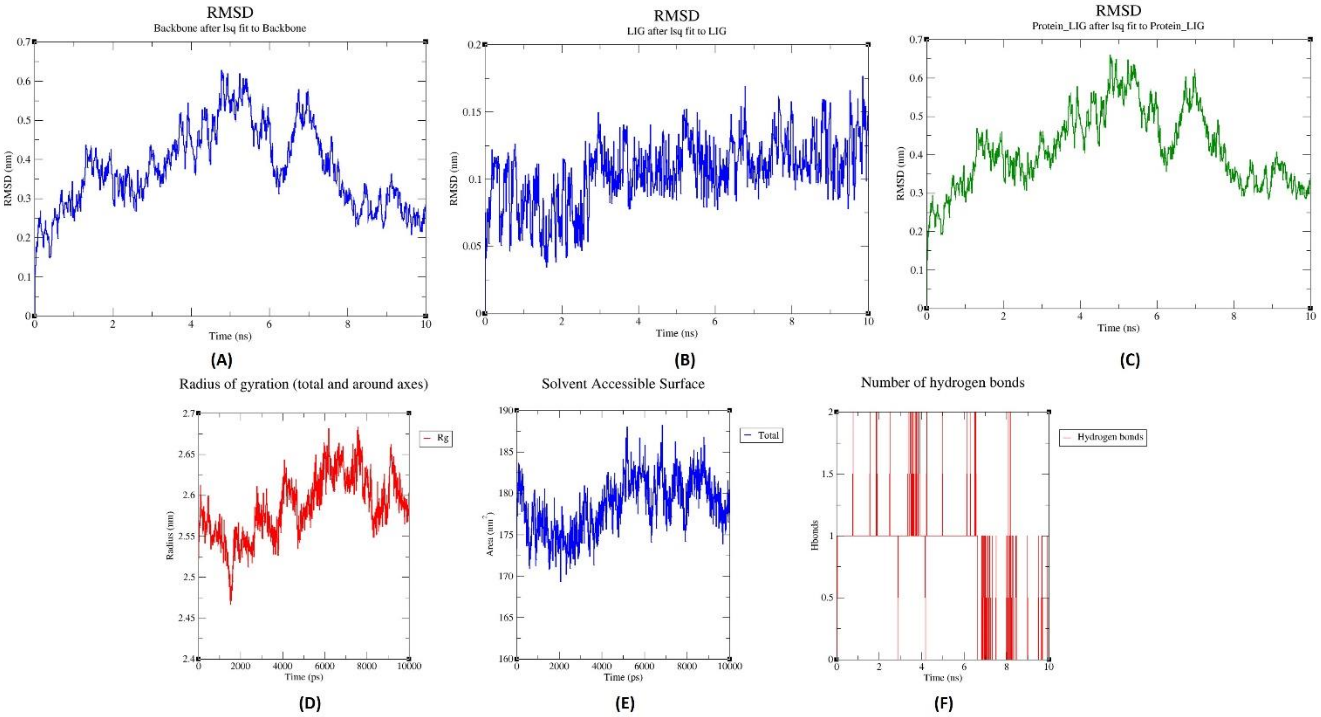

Appendix 10. Molecular dynamics simulation trajectories (10 ns) of the Jujubogenin–EGFR complex. (A) Protein backbone RMSD (lsq fit to backbone atoms), (B) ligand RMSD (lsq fit to ligand heavy atoms), (C) protein–ligand complex RMSD (lsq fit to protein–ligand heavy atoms), (D) radius of gyration (Rg) of the protein, (E) total solvent-accessible surface area (SASA), and (F) number of protein–ligand hydrogen bonds over the simulation time. All analyses were performed on the 10 ns production run using the Simulation Interaction Diagram module of Desmond. The data indicate initial relaxation within the first ~4 ns followed by stable equilibration with low ligand RMSD and persistent hydrogen bonding, confirming the stability of the docked Jujubogenin–EGFR complex.
