# Supplementary material for: Anticancer Effects of Ziziphus jujuba in Human Breast Carcinoma: An Integrated Study of Network Pharmacology, Molecular Docking and Dynamics Simulations and in-vitro Experimental Validation
Source: Iran J Pharm Res. 2026 Apr 20;25(1):e169917. doi: 10.5812/ijpr-169917 (PMC13558866; doi:10.5812/ijpr-169917)
Supplement: Supplementary file 1 [file ijpr-25-1-169917-s001.zip › Apendices 3-4.pdf]

Appendix 3. Physicochemical Properties of Selected Phytochemicals

Mol ID	MOL012940	MOL003410	MOL005360	MOL008034	MOL008647
Molecule Name	**Spiradine A**	**Jujubogenin**	**Malkangunin**	**Ceanothic Acid**	**Moupinamide**
MW	311.5	472.8	432.6	486.8	313.4
AlogP	1.24	4.39	1.84	5.36	2.86
Hdon	1	2	2	3	3
Hacc	3	4	7	5	5
OB (%)	113.52	66.95	57.71	73.52	86.71
Caco-2	0.29	0.49	0.22	-0.05	0.55
BBB	0.14	-0.1	-0.2	-0.5	-0.5
DL	0.6	0.6	0.6	0.8	0.3
FASA-	0.27	0.24	0.3	0.31	0.33
HL	15.7	10	4.09	14.2	3.71
Hepatotoxicity	No	No	No	No	No
Carcinogenicity	No	No	No	No	No
Cytotoxicity	No	No	No	No	No
Mutagenicity	No	No	No	No	No

Appendix 4. Molecular docking results for Jujubogenin against EGFR, HSP90AA1, and STAT3 using CB-Dock2.

Complex	CurPocket ID	Vina score	Cavity volume (Å^3)	Center (x, y, z)	Docking size (x, y, z)
**Jujubogenin-EGFR**	**C4**	-8.7	355	-61, 8, -33	23, 23, 23
**Jujubogenin-HSP90AA1**	**C1**	-7.5	1723	-8, -9, -24	23, 23, 23
**Jujubogenin-STAT3**	**C1**	-8.2	830	0, 25, 31	23, 23, 23
